# Dataset of authorship attribution from short-text multi topic representative in Bahasa Indonesia

**DOI:** 10.1016/j.dib.2026.112872

**Published:** 2026-05-20

**Authors:** Yohan Muliono, Ford Lumban Gaol, Andry Chowanda, Widodo Budiharto

**Affiliations:** a Computer Science Department, BINUS Graduate Program, Doctor of Computer Science Program, Bina Nusantara University, Jakarta 11480, Indonesia; b Computer Science Department, School of Computer Science, Bina Nusantara University, Jakarta 11480, Indonesia

**Keywords:** Authorship attribution, Stylometry, Indonesian language, Social media text, News articles, Text length analysis

## Abstract

Authorship attribution plays an important role in various applications, including digital forensics, Yauthorship verification, and the analysis of online textual content. While extensive benchmark datasets are available for high-resource languages such as English, publicly accessible datasets for authorship attribution in the Indonesian language remain limited. This limitation restricts systematic experimentation and comparative evaluation across different authorship attribution approaches in bahasa Indonesia.

To address this gap, this data article introduces an Indonesian authorship attribution dataset consisting of short-text documents collected from social media. Short-text data were obtained from Twitter using the official Academic API, The dataset covers three topical categories politic, financial, and mix and includes anonymized author labels to preserve authorship consistency without revealing personal identities.

In addition to the original text, the dataset provides explicit stylometric attributes, including word count and character count for each document, which are commonly used features in authorship attribution research. All data are consolidated into a single machine-readable CSV file to facilitate reuse, reproducibility, and further development of authorship attribution methods for the Indonesian language.

Specifications Table

**Table:** 

Subject	Computer Sciences
Specific subject area	***Authorship Attribution****:The task of identifying the author of a text based on distinctive writing style patterns rather than semantic content.*
Type of data	*Filtered Short-text (Twitter posts)*
Data collection	*Short-text data were collected when the official Twitter API was still publicly available, prior to the platform rebranding to X. Author accounts were identified through community input and public discussions on social media, targeting vocal and active writers to ensure sufficient data per author for authorship attribution. Data were retrieved using the official Twitter API. Data collection was conducted from late 2022 to early 2023. Author selection required a minimum of 1000 tweets prior to filtering; after preprocessing, each retained author maintained a minimum of 600 tweets. The dataset comprises 54 authors in total (22 Financial, 17 Politic, 15 Mix), with an average of 1567 tweets per author (minimum: 606, maximum: 5652). Retweets were excluded as they represent the words of other users rather than the author’s own writing. Author selection was based on the predominant topic of their Twitter activity, with each author manually assigned to the category Politic, Finance, or Mix that best reflected the majority of their posted content.*
Data source location	*Bina Nusantara University*
Data accessibility	Repository name: Mendeley Data Data identification number: 10.17632/3z4rn8frsz.2 Direct URL to data: https://data.mendeley.com/datasets/3z4rn8frsz Instructions for accessing these data: From the URL, there will be a CSV, all the data will be there
Related research article	*N/A*

## 1 Value of the Data

• These data provide a dedicated dataset for authorship attribution in the Indonesian language, a research area widely studied in high-resource languages such as English but under-represented for many others. The limited availability of such resources has constrained research progress in low- and medium-resource languages; this dataset addresses that gap by offering curated short-text data suitable for stylometric analysis.
• These data enable multi-topic analysis within short-text environments, comprising writings drawn from political, financial, and mixed-content discussions. This structure allows researchers to examine how writing style varies across subject matters a well-known challenge in authorship attribution and to investigate topic-induced stylistic variation and its impact on model robustness. Explicit length-based features (word count and character count) are included to facilitate direct comparison with prior studies in other languages and to support both traditional stylometric and modern machine learning approaches.
• These data can serve as a benchmark for evaluating and comparing authorship attribution models in Indonesian, including transformer-based approaches such as IndoBERT, supporting systematic and reproducible model comparison across methods.
• These data are relevant for forensic and security-oriented research, as authorship attribution is not limited to author identification in academic settings. The dataset can support studies related to digital forensics [1], online behavior analysis [2], and attribution of anonymous or disputed texts, which are increasingly important in addressing the spread of hoaxes and harmful content in digital environments [3]. @mentions and URLs were removed during preprocessing, the retained stylometric signals including writing length patterns, function word usage, and lexical distributions remain informative for authorship-based attribution. These features are author-intrinsic and do not depend on referential metadata, making the dataset applicable to forensic scenarios where identifying the origin of anonymous or pseudonymous content is required.
• These data support reproducibility and methodological transparency, as they are organized in a structured, machine-readable format and can be easily integrated into existing experimental pipelines. Note that Indonesian stopwords are not provided as a separate column in the dataset; users are expected to supply their own stopword list. Common resources for Indonesian stopword lists include PySastrawi and NLTK Indonesian corpora.
• Several potentially confounding features were removed to improve the reliability of the authorship attribution analysis, including extra spaces, URLs, and retweet markers. In particular, retweets were excluded because they represent the words of other users rather than the author’s own writing, which could introduce stylistic noise and distort the attribution process.

## 2 Background

The dataset was compiled in response to the limited availability of publicly accessible resources for authorship attribution in the Indonesian language. While extensive benchmark datasets and baselines exist for high-resource languages such as English [4], comparable datasets for Indonesian remain scarce. This limitation has hindered systematic evaluation and methodological comparison across different authorship attribution approaches. Since short-text [5,6] analysis in Authorship Attribution is emerging in several languages [4,[7], [8], [9], [10], [11]]

From a methodological perspective, authorship attribution research relies heavily on stylometric features that capture stable writing characteristics independent of semantic content. Previous studies in other languages have demonstrated the importance of text length, structural features, and topic variation in authorship attribution experiments. However, datasets that explicitly support these methodological requirements are not widely available for Indonesian.

This data article complements related authorship attribution research by providing the underlying dataset used for model development and evaluation. By publishing the dataset separately, this work enables transparency, reproducibility, and reuse of the data for alternative modeling approaches, feature engineering strategies, and comparative studies. The dataset is intended to serve as a reusable resource for future research in authorship attribution, stylometry, and related forensic text analysis [1] tasks involving Indonesian-language text.

## 3 Data Description

The dataset consists exclusively of short-text documents designed for authorship attribution research. All documents are stored within a single CSV file and are systematically organized in a structured, machine-readable format. Each document is annotated with topical labels and stylometric attributes required for authorship attribution analysis, as summarized in Table 1. A detailed description of each data component is provided below. The dataset comprises 84,653 documents produced by 54 authors in total, distributed across three topic categories: Mix (40,305 documents, 15 authors), Financial (24,935 documents, 22 authors), and Politic (19,413 documents, 17 authors). The Label column contains anonymized author identifiers, assigned as sequential numeric codes replacing original usernames to ensure no personally identifiable information is retained in the dataset.

**Table 1:** Attributes included in the dataset.

Attribute	Description
Text	Original Indonesian text
Label	Anonymized author identifier
topic	Topic category (politic, financial, mix)
type	Text type indicator (short-text)
Word_Count	Number of words in the document
Char_Count	Number of characters in the document

**Short-text**: Short-form Indonesian text collected from social media, characterized by concise length and informal writing style. These texts typically contain limited word and character counts and reflect spontaneous author behaviour.

**Politic**: Texts discussing political topics, including government, public policy, political actors, and political events.

**Financial**: Texts focusing on financial and economic topics, such as markets, investments, economic conditions, and financial news.

**Mix**: Texts produced by authors who write across multiple topics, not limited to author with specific content within their writing history.

**Word count**: The total number of words contained in each document, provided as a length-based stylometric feature commonly used in authorship attribution studies.

**Character count**: The total number of characters contained in each document, capturing structural writing patterns at the character level.

Text length categories are defined and summarized in Table 2.

**Table 2:** Word and character length distribution.

Text type	Topic	Word count (min–max)	Character count (min–max)	Average word count	Average character count	Number of documents
Short-text	Finance	1 – 66	1 – 280	17	108	24,935
Short-text	Politic	1 – 54	1 – 280	21	152	19,413
Short-text	Mix	1 – 30	1 – 140	10	64	40,305
Short-text	Total	1 – 66	1 – 280	14	95	84,653

Several authorship attribution studies incorporate word length and character length as features [11,10] to represent structural writing patterns that are independent of semantic content. Table 2 provides a detailed distribution of word and character lengths across each topic category, including minimum, maximum, and average values, along with the number of documents and Table 3 summarizes the distribution of authors and documents across each topic category, including the minimum, average, median, and maximum number of documents per author. These features are commonly used to support the analysis of writing style consistency across documents produced by the same author.

**Table 3:** Document distribution per topic.

Topic	Number of authors	Number of documents	Min docs per author	Avg docs per author	Median docs per author	Max docs per author
Finances	22	24,935	606	1133.41	1183	1984
Politic	17	19,413	608	1141.94	1199	2012
Mix	15	40,305	1523	2687.00	2667	5652
Total	54	84,653	606	1567.65	1198.5	5652

Table 4 presents three example rows drawn from the dataset, one from each topic category. The Politic example reflects the typical short, event-driven commentary style found in political tweets, written entirely in Bahasa Indonesia. The Finance example illustrates the code-mixed Indonesian-English writing style characteristic of financial authors in the dataset, with domain-specific vocabulary such as *return* and *investasi*. The Mix example demonstrates why certain authors are assigned to this category the text spans both social commentary and financial themes, reflecting the multi-topic nature of the author's writing history. Together, these examples show the structure of the CSV file and the range of writing styles captured across the three topic categories.

**Table 4:** Example rows for the dataset.

Text	Label	topic	type	Word_Count	Char_Count
70 ribu pengunjung akan hadir memadati GBK. Insya Allah membawa berkah lapangan kerja untuk UMKM di sekitar kawasan GBK	author_1	Politic	short-text	19	119
One day, kita semua bakal sadar; bahwa yang terpenting dalam investasi itu, bukan return setinggi-tingginya, tapi konsisten investasi & sustainable return.	author_18	Finance	short-text	21	155
Melihat fenomena Ghozali, jadi tersadar jaman udah berubah banget. Dulu orang-orang invest di emas, sekarang invest di foto emas-mas.	author_44	Mix	short-text	19	133

Previous studies have reported that text length can influence authorship attribution performance [6], with longer texts often providing more stable stylistic signals for author identification. As a result, word count and character count are frequently included as supporting stylometric features in authorship attribution research. In this dataset, explicit word and character length attributes are provided to facilitate length-aware analysis and to support researchers conducting authorship attribution experiments in the Indonesian language.

Fig. 2 shows the 20 most frequent words in the dataset when Indonesian stopwords are retained and removed. The with-stopwords distribution reveals the dominance of high-frequency function words such as *yang, di*, and *dan* which are author-intrinsic and largely topic-independent. In authorship attribution research, function word frequencies are among the most reliable stylometric features because they reflect subconscious writing habits that remain stable across different topics and are difficult to deliberately manipulate [12]. The without-stopwords distribution exposes the content-bearing vocabulary of the dataset, showing the dominant thematic terms after functional noise is removed. Comparing both distributions helps researchers decide which feature sets are most appropriate for attribution tasks on this dataset.

Meanwhile Fig. 3 represents the top 20 most frequent words in the dataset with Indonesian stopwords retained for a stylometric analysis perspective, usage of stop words could be considered as one of strong stylometry feature [12]. Word frequencies are shown separately for financial, politic, and mix topics, allowing comparison of lexical distributions across topics and text types.

## 4 Experimental Design, Materials and Methods

The dataset was constructed using a multi-stage data acquisition and processing pipeline designed to collect, filter, and organize Indonesian-language text for authorship attribution experiments. The overall workflow is illustrated in Fig. 4 and consists of data acquisition, text filtering, feature extraction, and dataset consolidation.

### 4.1 Data sources and acquisition

Data were collected from Twitter using the official Twitter Academic API during the period from late 2022 to early 2023, prior to the platform rebranding to X. Author accounts were identified through public social media discussions by querying for individuals recognized as active and vocal writers. An author was considered active if they had published a minimum of 1000 tweets prior to filtering; after preprocessing, each retained author was required to have at least 600 remaining tweets. The resulting dataset contains 54 authors with an average of 1567 tweets per author (minimum: 606, maximum: 5652). Texts were retrieved programmatically using Python-based scripts that interacted directly with the Twitter API. Note that these scripts were developed for the Twitter Academic API and may not be compatible with the current X (formerly Twitter) API; they are available from the corresponding author upon request.

### 4.2 Text filtering and pre-processing

All collected texts underwent a filtering process to standardize the content prior to dataset integration. The filtering stage included the following steps:

• Removal of user mentions from social media texts.
• Removal of URLs and hyperlinks.
• Removal of extra whitespace and formatting artifacts.
• Removal of retweets, as they represent the words of other users and would introduce stylistic noise into the authorship attribution analysis.

These steps were applied across the data to ensure uniform text formatting. Hashtags and emojis were retained in the stored text, as they may carry author-specific stylistic signals relevant to authorship attribution. No language normalization was applied, including no lowercasing, spelling normalization, or informal-to-formal word mapping, in order to preserve the natural stylometric properties of each author's writing. Tokenization was performed using whitespace splitting, consistent with the word count computation. Stopwords were not removed from the stored text; the stopword-filtered word frequency analysis shown in Fig. 2, Fig. 3 is provided for descriptive purposes only and does not reflect any preprocessing applied to the dataset.

### 4.3 Text categorization and labeling

Each document was assigned an anonymized author label to preserve author consistency while preventing disclosure of author identity. Documents were categorized into one of three topical labels: politic, financial, or mix. Topic categorization was performed manually by the authors based on the predominant subject matter of each author’s collected posts. The mix category was assigned to authors whose collected texts spanned more than one topic domain.

### 4.4 Feature extraction

Two length-based stylometric features were computed for each document: word count and character count. Word count was calculated as the total number of whitespace-separated tokens using Python's built-in str.split() function, while character count was calculated as the total number of characters using Python's built-in len() function. Both features were computed using custom Python scripts leveraging the pandas library for data processing, and stored explicitly as dataset attributes in the final CSV file. While additional stylometric features were considered during dataset development, including character n-grams, function word frequencies, and punctuation-based features, only word count and character count are provided as pre-computed attributes in this release. Researchers wishing to extract richer stylometric feature sets may derive them directly from the raw text column included in the dataset.

### 4.5 Dataset final output

**Fig. 1:**
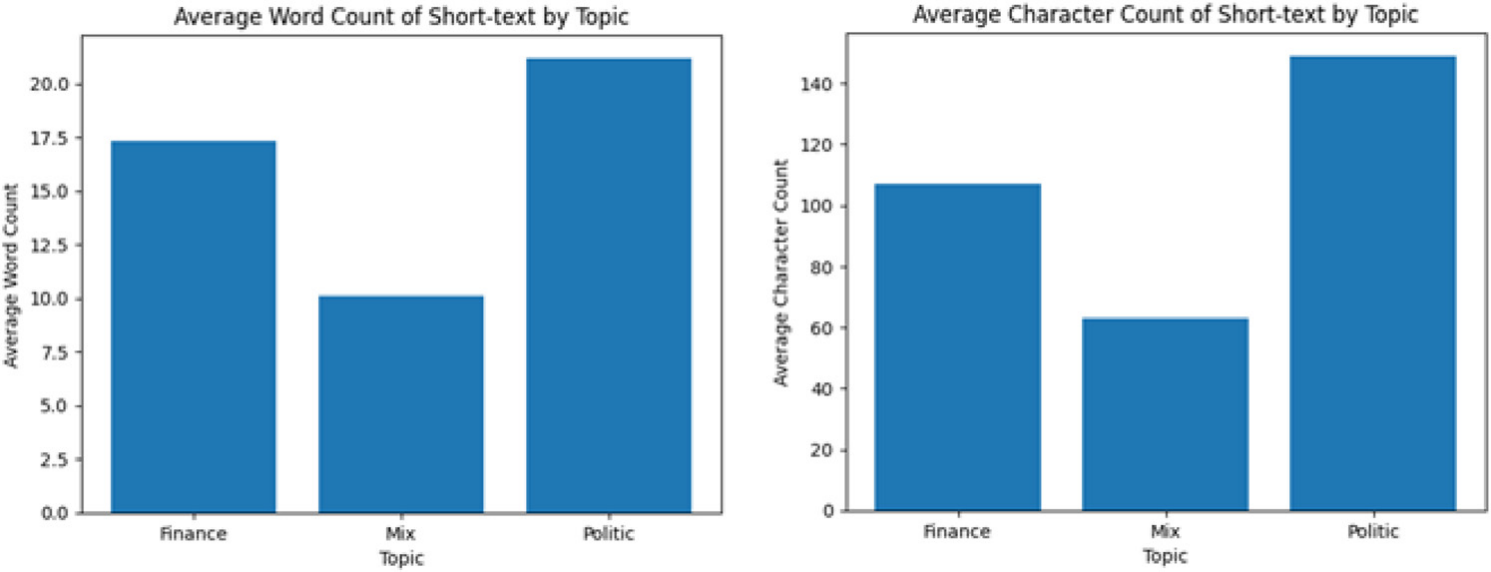
Distribution of average word and character of each topic.

**Fig. 2:**
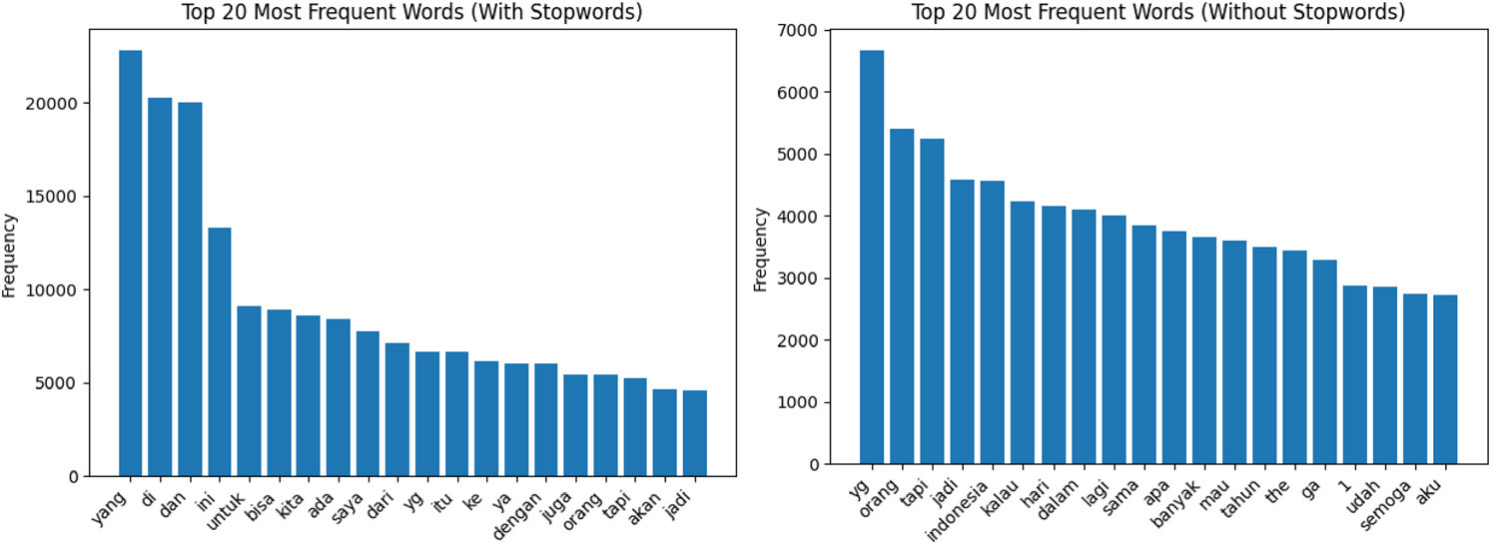
Top 20 most frequent words used in the dataset with and without stopwords.

**Fig. 3:**
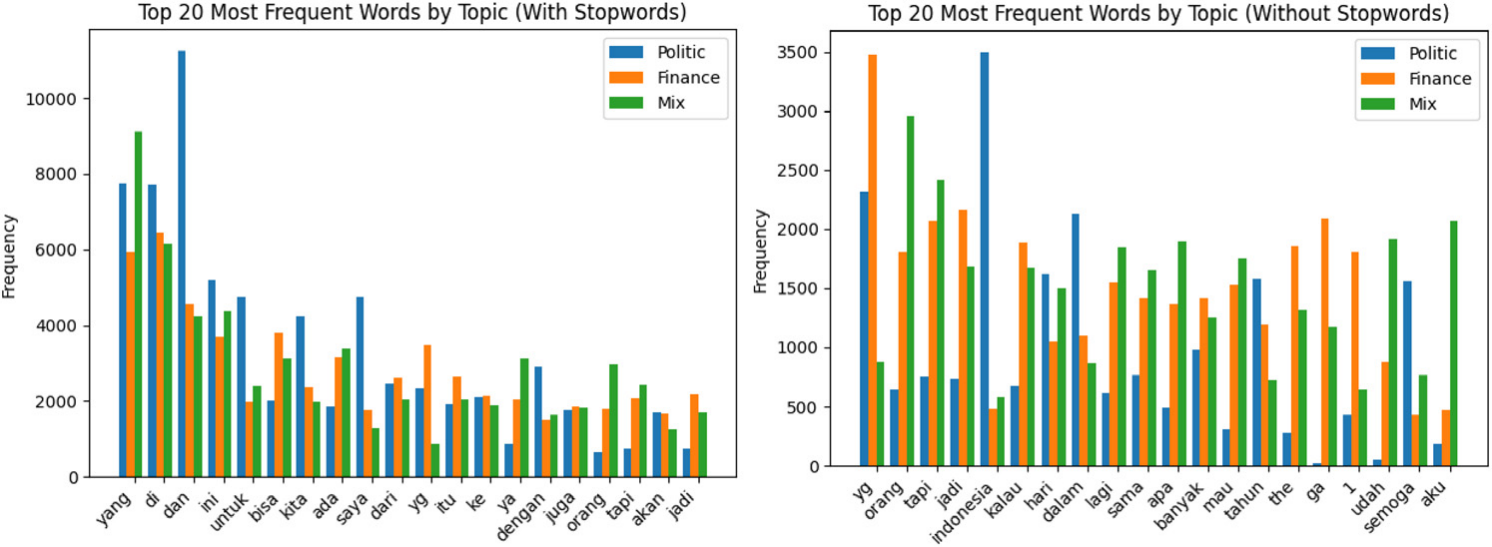
Top 20 most frequent words used in the dataset with and without stopwords per topic.

All figures presented in this article were generated manually using Microsoft Excel, based on aggregated statistics computed from the CSV dataset. The word frequency distributions shown in Fig. 2, Fig. 3 were derived by calculating term frequencies across all documents and per topic category respectively, with stopword filtering applied separately for the comparative visualisations. The word and character length distributions shown in Fig. 1 were derived from the Word_Count and Char_Count columns already present in the dataset. No separate intermediate files were produced; all figures can be reproduced directly from the published CSV file using standard spreadsheet or data analysis tools.

**Fig. 4:**
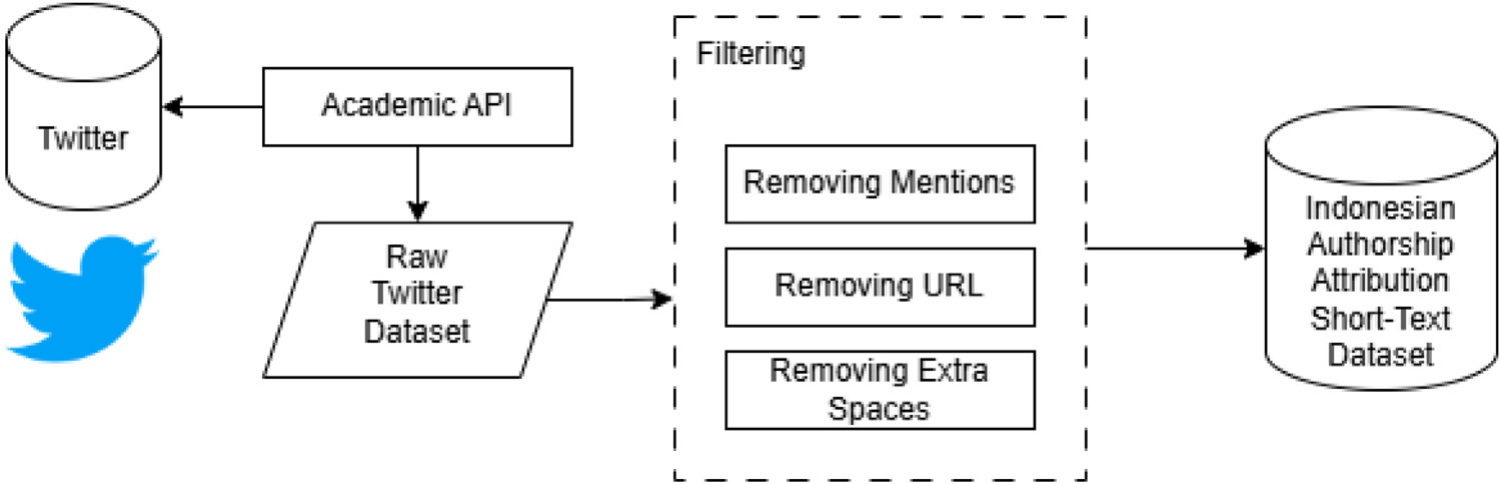
Activity flow of collecting indonesian authorship attribution dataset.

## Limitations

The dataset is limited to Indonesian-language short texts collected from Twitter between late 2022 and early 2023, and may not generalise to other platforms, time periods, or longer text formats. Author selection was based on community-identified active writers rather than random sampling, which may introduce selection bias and limit the representativeness of the authorship styles captured. In particular, the dataset skews toward authors who are publicly active and vocal on political and financial topics on Twitter, which may not capture the full stylometric diversity of Indonesian-language writers across other demographics, platforms, or writing domains. Only word count and character count are provided as pre-computed stylometric features; other commonly used features (e.g., character n-grams, function word frequencies) must be derived by the user. The data collection scripts were developed for the Twitter Academic API and may not be compatible with the current X (formerly Twitter) API.

## Ethics Statement

This study involved data collected from publicly available social media content. No informed consent was required, as all data were obtained from publicly accessible posts. All author identities were fully anonymized, and no personal, sensitive, or private information was retained in the dataset. Usernames, mentions, and other identifiable metadata were removed during the data processing stage.

Data collection and redistribution were conducted in compliance with the data usage and redistribution policies of the Twitter platform that were applicable at the time of data acquisition. Only textual content and derived features are provided in the dataset, without including user identifiers or platform-specific metadata.

## CRediT Author Statement

**Yohan Muliono**: Conceptualization, Methodology, Data Curation, Software, Original draft prepa- ration, Visualization. **Ford Lumban Gaol**: Supervision, Validation. **Andry Chowanda**: Supervise, validate, write, review, and edit. **Widodo Budiharto**: Supervision, Validation, Investigation. All authors read and approved the manuscript.

## Data Availability

Mendeley DataMulti Topic Short-Text Authorship Attribution Data in Bahasa Indonesia (Original data)
